# Gravity-driven postseismic deformation following the Mw 6.3 2009 L’Aquila (Italy) earthquake

**DOI:** 10.1038/srep16558

**Published:** 2015-11-10

**Authors:** Matteo Albano, Salvatore Barba, Michele Saroli, Marco Moro, Fabio Malvarosa, Mario Costantini, Christian Bignami, Salvatore Stramondo

**Affiliations:** 1 National Institute of Geophysics and Volcanology, National Earthquake Center, Rome, 00143, Italy; 2 University of Cassino and Southern Lazio, Department of Civil and Mechanical Engineering, Cassino, 03043, Italy; 3 e-GEOS, an Italian Space Agency (ASI) and Telespazio Company, Rome, 00156, Italy

## Abstract

The present work focuses on the postseismic deformation observed in the region of L’Aquila (central Italy) following the Mw 6.3 earthquake that occurred on April 6, 2009. A new, 16-month-long dataset of COSMO-SkyMed SAR images was analysed using the Persistent Scatterer Pairs interferometric technique. The analysis revealed the existence of postseismic ground subsidence in the mountainous rocky area of Mt Ocre ridge, contiguous to the sedimentary plain that experienced coseismic subsidence. The postseismic subsidence was characterized by displacements of 10 to 35 mm along the SAR line of sight. In the Mt Ocre ridge, widespread morphological elements associated with gravitational spreading have been previously mapped. We tested the hypothesis that the postseismic subsidence of the Mt Ocre ridge compensates the loss of equilibrium induced by the nearby coseismic subsidence. Therefore, we simulated the coseismic and postseismic displacement fields via the finite element method. We included the gravitational load and fault slip and accounted for the geometrical and rheological characteristics of the area. We found that the elastoplastic behaviour of the material under gravitational loading best explains the observed postseismic displacement. These findings emphasize the role of gravity in the postseismic processes at the fault scale.

The Italian peninsula is the result of the back-arc opening of the Tyrrhenian Sea and the flexural hinge retreat of the Adria plate since the Neogene period^1^,^2^. Since the Pleistocene, a NE-trending extensional stress field, mainly concentrated along the axial belt, has been superimposed onto the crustal shortening and thrusting^3^,^4^. Currently, this extension persists in the axial belt, whereas compression is present to the east, as evidenced by focal mechanisms, geodetic data, and borehole breakouts^5^,^6^,^7^,^8^,^9^,^10^. Consequently, normal faults in the Apennine axial belt dissect the Pliocene and Quaternary continental deposits^11^,^1^ and bound graben and half-graben basins in places (Fig. 1a). These normal faults are often capable of generating earthquakes and are mapped in several databases^12^.

On April 6th, 2009, a normal faulting earthquake of Mw 6.3 hit the central Apennines, causing severe damage to the city of L’Aquila and neighbouring villages. The earthquake occurred along the axial belt in an area where historical earthquakes are known to be large (e.g., year 1349: Mw 5.9; 1461: Mw 6.4; Feb. 2, 1703: Mw 6.7; and 1915: Mw 7.0)^13^. Paleoseismological data indicate inter-event times for Mw > 6.5 earthquakes of 1–2 kyr, corresponding to possible vertical throw rates of 0.4–1.2 mm/yr^14^,^7^.

The mainshock nucleated at a depth of approximately 9 km, with a hypocenter 4 km southwest of the city of L’Aquila (Fig. 2a). The 2009 L’Aquila earthquake dislocated the Paganica fault (PF in Fig. 2a), a normal fault trending NW-SE and dipping 45–50 ° to the SW^15^,^16^,^17^. The fault exhibits throw in the Holocene sediments of more than 0.25 m^18^. The mainshock was preceded by foreshocks of up to Mw 4.3 and was followed by a 3-year-long aftershock sequence comprising more than 80,000 events, among which seven were Mw > 5. These aftershocks occurred within a 35-km-long NW-SE-trending area^19^,^20^. This long seismic sequence provided a significant amount of data, which allowed to investigate the seismic source and surface effects^21^. Numerous studies have investigated the coseismic displacement field. The surface displacements came from GPS networks^22^ and SAR Interferometry (InSAR)^23^,^19^. The InSAR data^19^ highlight a maximum Line of Sight (LOS) lengthening of approximately 25 cm west of the Paganica fault, which emphasizes the subsidence of the hanging wall (contour lines in Fig. 2a). To the east of the Paganica fault, a rather uniform LOS lengthening of approximately 10 cm is observed, indicating that the eastward component of motion is larger than the footwall uplift.

The coseismic displacement field was simulated in a homogeneous elastic medium using finite-fault dislocation models^19^,^24^ and in a heterogeneous medium using finite element models^25^,^26^. These joint inversion studies retrieved a coseismic fault slip distribution with peak values of 90–100 cm over a fault plane of approximately 100 km^2^ (Fig. 2a). In constraining the inversion procedure, the choice of datasets causes certain differences in the retrieved slip distribution, indicating a non-optimal selection of smoothing parameters^27^.

The postseismic displacement field, based on GPS, InSAR, and high-precision levelling data, shows two maxima located NE and SW of the town of L’Aquila, adjacent to but not coincident with the coseismic subsidence^28^,^22^,^29^,^30^. To retrieve the slip distribution, the authors of these studies applied methods and model parameterizations that were inherited from the coseismic studies. In particular, the geodetic and satellite data were modelled by linear inversion in a homogeneous elastic medium assuming that the coseismic fault continues to slip during the postseismic phase.

The calculated slip distributions exhibit substantial differences depending on the choice of the inversion procedure and the model parameters. In fact, an inversion that used the simulated annealing technique with 200 days of GPS data calculated three postseismic slip patches, which did not coincide with the maximum coseismic slip at depth^22^. Later, the same authors performed a linear elastic joint inversion of GPS, InSAR, and levelling data using a bounded-value least squares algorithm and found similar results^28^,^29^. An inversion that was based on the principal component analysis of 300 days of GPS data calculated two postseismic slip patches that were located at the middle of the fault^30^. The difference in the number or shapes of the retrieved slip maxima indicates several uncertainties in the data or model parameterization^27^.

All these studies ruled out non-tectonic causes of the observed postseismic displacement. In fact, certain authors note that ground subsidence occurs regardless of the lithologies and topographic elevations^28^. These studies, however, consider only surface processes, such as landslides and sediment compaction, to be non-tectonic but neglect the rheological behaviour of the medium. In fact, plastic deformation has not been explored for the L’Aquila earthquake.

Tectonic phenomena, e.g., the slip along fault planes, may not be the only causes of ground deformation. In fact, many phenomena contribute to ground deformation, including the visco-elastic relaxation in the lower crust and upper mantle caused by the stress perturbation induced by the mainshock^31^, the poro-elastic rebound due to the pore pressure and fluid flow changes after the earthquake^32^, the variation of the phreatic level of Quaternary soft deposits^33^, the compaction induced by the liquefaction of buried sandy deposits^34^, the slope movements induced by gravity^35^, the redistribution of local stress, and plasticity. These phenomena all play different roles depending on the geological, geotechnical, and hydrogeological properties of the earthquake area.

In the coseismic phase, tectonic deformation is one order of magnitude larger than the non-tectonic deformation. For this reason, the coseismic surface deformation is modelled with linear and nonlinear inversion schemes that neglect non-tectonic phenomena in favour of fault slip. Conversely, the non-tectonic phenomena influence the postseismic phase. In fact, tectonic and non-tectonic displacements may reach similar values and affect the same or nearby locations. Discriminating between tectonic and non-tectonic phenomena requires modelling every phenomenon affecting the surface deformations. Thus, studying non-tectonic processes requires methods that differ from those used in the coseismic studies.

Unlike linear inversion schemes, finite element methods can model the different phenomena that characterize the postseismic phase. Indeed, the rate of displacement associated with each single phenomenon depends on the topography and the rheology of the geomaterials.

The postseismic displacement of the 2009 L’Aquila earthquake occurred primarily in carbonatic and coarse-grained deposits. These deposits are not very susceptible to compaction induced by liquefaction or groundwater changes. In the surrounding area, ground deformation phenomena, such as slope movements, may have been caused by historical earthquakes in the central Apennines^36^. This idea was corroborated by the 2009 mainshock, which activated slope movements in the area^37^.

In this work, we performed an elastoplastic modelling of the postseismic displacements due to plastic strains driven by gravity. We employed bidimensional finite element models and original satellite data. In particular, we focused on the surface displacements measured by COSMO-SkyMed (hereafter CSK) InSAR data covering 16 months after the mainshock. We applied a multi-temporal InSAR technique known as PSP-InSAR^38^, which produced a surface velocity map and relative deformation time series at each coherent scatterer.

Particular emphasis has been placed on the deformation pattern detected on the Mt Ocre ridge, southwest of the town of L’Aquila (Fig. 2b). This area is characterized by numerous landforms that are primarily oriented parallel to the ridge axis (NW-SE). These morphological features are typically associated with surface deformation processes induced by gravity, such as double crest lines, scarps, and trenches^39^. Such gravitational processes are accelerated by large earthquakes^40^. Indeed, the study area exhibits a previously unexplained anomaly in the postseismic deformation that is not ascribable solely to the coseismic source.

The observed postseismic surface displacements were modelled in two sections across the Mt Ocre ridge. The results of the numerical models indicate that a significant proportion of the plastic deformation in the ridge was initiated by the occurrence of the mainshock but was driven by gravity.

## Results

### InSAR data processing and interpretation

**Figure 1:**
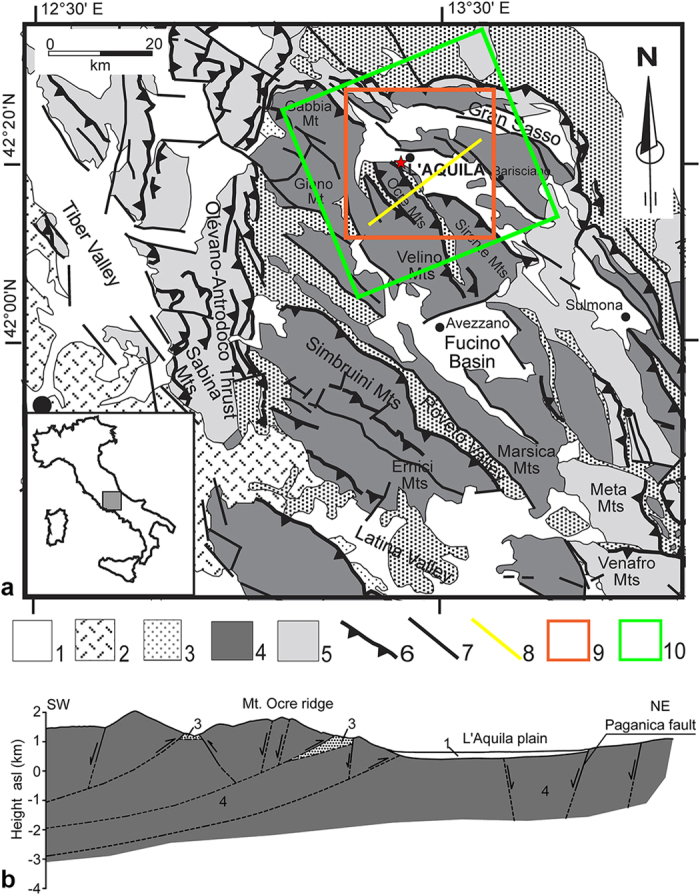
(**a**) Simplified geological and structural map of the central Apennines. Key to the legend: 1) marine and continental clastic deposits (Pliocene-Quaternary); 2) volcanic deposits (Pleistocene); 3) syn-orogenic, hemipelagic, and turbiditic sequences (Tortonian-Pliocene); 4) carbonate platform deposits (Triassic-Miocene); 5) slope and pelagic deposits (Liassic-Miocene); 6) main thrust; 7) main normal and/or strike-slip fault; 8) trace of the geological sketch presented in panel b; 9) earthquake study area; 10) extent of the SAR images. The red star indicates the epicentral location of the 2009 Mw 6.3 earthquake. (**b**) Geological sketch^42^,^43^,^44^,^45^. Map was created with Adobe Illustrator software.

To study the temporal evolution of the displacements in the postseismic phase, we used a set of 35 SAR images that cover an area of 40 × 40 km^2^ (Fig. 1a) and span approximately 16 months, from April 12, 2009 to August 6, 2010. These images were acquired by the CSK constellation on its ascending orbit in stripmap mode at a 40 ° incidence angle. We analysed the SAR images by applying the Persistent Scatterer Pair technique (PSP)^38^. This technique allowed us to estimate the displacement time series in a dense set of locations corresponding to the identified persistent scatterers (supplementary Fig. S1), considering the first April 12, 2009 CSK acquisition as our reference epoch.

Figure 3a shows the postseismic displacement field 16 months after the mainshock, expressed in terms of the satellite’s LOS. The postseismic displacement map exhibits two areas of significant subsidence. In the northern section of the Paganica fault, the hanging wall shows a maximum LOS subsidence of 40 mm. The subsidence is rather fast, with 50% of the total deformation occurring within the first three months and 90% within seven months (point 2 in the supplementary Fig. S2). The maximum is located within 2–3 km of the Paganica fault line, suggesting a shallow or near-surface origin. This hypothesis is confirmed by the occurrence of many surficial aftershocks in the subsided area^41^ (supplementary Fig. S2a), generated by the shallow slip of the Paganica fault^28^ or unmapped neighbouring structures.

The second subsidence area is primarily on the Mt Ocre ridge (SW of L’Aquila) and covers an area that is 5–6 km wide (Fig. 3a and the detailed area in Fig. 3b). This long-wavelength signal reaches maximum values between 10 and 30 mm (point 1 in the supplementary Fig. S2).

The cumulated LOS displacement profiles along two sections perpendicular to the ridge axis exhibit several interesting features (Fig. 4a). In particular, a 3–4-km-long convex displacement pattern is located in the high-relief zone of the Mt Ocre ridge (blue-sided arrows). In this area, the displacement is larger than that on the L’Aquila plain. Furthermore, Fig. 5a shows that the displacement time series are nonlinear in the high relief zone of the Mt Ocre ridge (points 2A and 2B), almost linear on the flat or gently sloping ground on the L’Aquila plain (points 3A, 4A, and 4B), and close to zero far from the Paganica fault (points 1A and 1B).

The nonlinear temporal evolution and the overall spatial shape of the postseismic displacement on the Mt Ocre ridge cannot be attributed solely to the slip associated with the coseismic source. Instead, the nonlinearity in the time series suggests that another deformation mechanism has to be considered during the postseismic phase. Given the morphological features characterizing the Mt Ocre ridge, we hypothesized that part of the observed deformation results from the gravitational spreading of the ridge, induced by the ground shaking^40^. Coseismic subsidence caused instability in the ridge, triggering plastic deformation and local landslides that produced excess deformation with respect to the L’Aquila plain.

### Numerical analysis

To quantify the role that gravity had on the postseismic displacement across the Mt Ocre ridge, we developed a numerical model.

The NW-SE alignment of the geomorphological features of the Mt Ocre ridge suggests that the opening of the ridge is facilitated, whereas the along-ridge component of displacement is inhibited. To take advantage of the symmetry in the displacement, we considered sections A and B to be orthogonal to the Mt Ocre ridge axis, and we constructed two 2D plane-strain finite element models (Fig. 4b).

The model includes the topography of Mt. Ocre and the L’Aquila plain and extends to a depth of approximately 4 km. These depths are nearly unaffected by the aftershock distribution; 84% of the earthquakes, including all of the largest earthquakes, are deeper than 4 km^41^ (supplementary Fig. S3).

**Figure 2:**
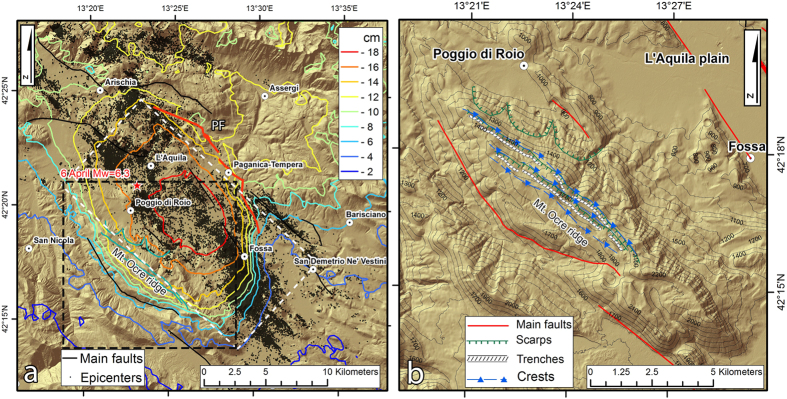
(**a**) Detail of the study area (red box in Fig. 1a). The red star represents the location of the mainshock, and the black dots indicate aftershocks of Mw < 5. The contours identify the line-of-sight coseismic displacement pattern from the InSAR data^19^. The white rectangle indicates the surface projection of the Paganica fault (PF red line). (**b**) Detail of the Mt Ocre ridge area (black box in Fig. 2a) Maps were created with ArcGIS and Adobe Photoshop software.

**Figure 3:**
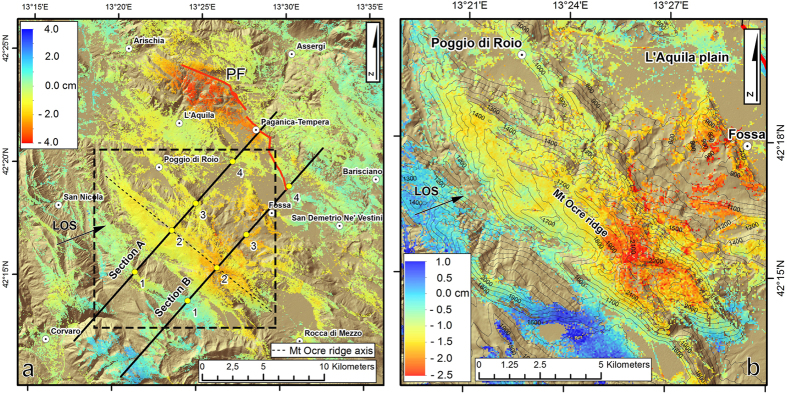
(**a**) Line-of-sight postseismic displacement map from the InSAR CSK dataset for the time interval between April 12, 2009 and August 6, 2010. (**b**) Detail of the postseismic displacement affecting the Mt Ocre ridge area (black box in Fig. 3a). Maps were created with ArcGIS and Adobe Illustrator software.

**Figure 4:**
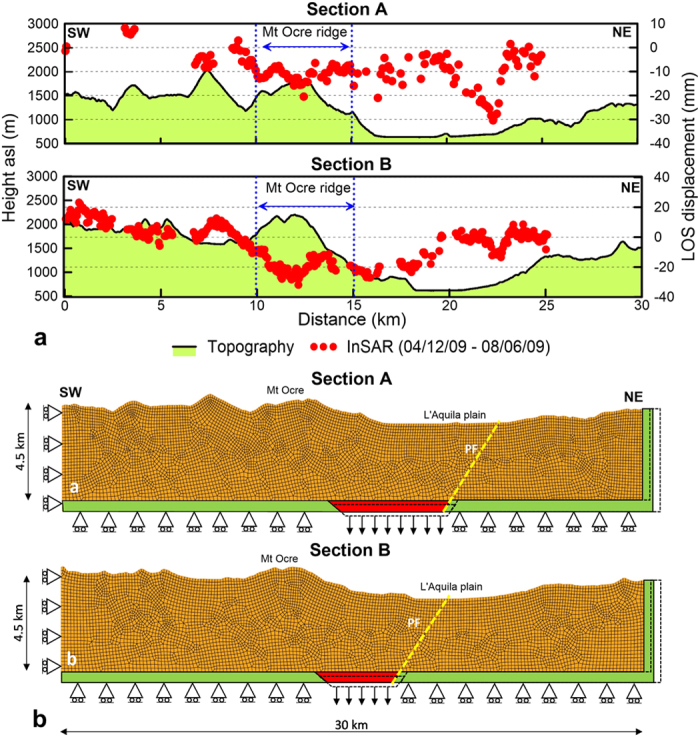
(**a**) Postseismic (August 6, 2010) displacement profiles for sections A and B (section traces are shown in Fig. 3a). (**b**) Finite element models for sections A and B (Fig. 3a). The yellow line identifies the projection of the Paganica fault (PF). The rollers constrain zero displacement normal to the boundary. The red and green bars are infinitely stiff elements; the red wedge simulates the coseismic and postseismic dislocations by moving downwards, while the green rigid support moves to the right. The dashed lines indicate the positions of the rigid elements at the end of the analysis (not to scale). Figures were created with Golden Software Grapher and Adobe Photoshop.

The boundary conditions include zero horizontal displacements at the edges. The two modelled sections (Fig. 2a) are defined such that the SW edges correspond to zero observed LOS displacement. The location of the NE edge is selected so that the model’s boundary is far from the Paganica fault and does not affect the modelled displacements. An infinitely stiff plate bounds the model at the bottom (red and green elements in Fig. 4b). A wedge is used to simulate the coseismic slip and afterslip of the master fault.

The model is composed of a homogeneous and isotropic material. The geological sections that are available for the study area report relatively homogeneous lithologies that are mainly composed of undifferentiated calcareous and dolomitic deposits that are Cretaceous to Late Triassic in age^42^,^43^,^44^,^45^. At depth, these lithologies have similar stiffnesses and strengths^46^. The modelled rock volume is highly jointed, and the mapped fractures are significantly less than l km long^40^. Thus, we used the equivalent continuum approach in the modelling. In several similar field cases, the equivalent continuum analysis was used to satisfactorily model the behaviour of a fractured rock mass as a whole using equivalent material properties^47^.

To evaluate the effect of the plasticity on the computed ground displacements, two isotropic constitutive models, one linear elastic (ELA) and one linear elastic-perfectly plastic (PLA), were tested by performing independent analyses.

The coseismic and postseismic displacements were simulated following three different stages. In the first stage, the gravity load is applied to bring the model to an initial, physically realistic equilibrium state. During this stage, the horizontal and vertical displacements along the lower boundary plates are fixed (including the wedge), only vertical displacements are allowed at the lateral edges, and the surface of the model is free.

In the second stage, the coseismic displacement is simulated by moving the wedge downward, without changing the remaining boundary conditions and loads. The vertical displacement of the rigid wedge is set to fit the LOS coseismic displacement profiles.

In the last stage, we simulated the time evolution of the postseismic displacement. To simulate the afterslip on the Paganica fault, the rigid wedge is moved with a constant downward velocity for 16 months, the time span of the CSK images. Typically, the afterslip varies with time because it depends on the aftershocks and the rheology of the materials. However, assuming a constant velocity highlights the role of tectonics and gravity without introducing further complications; evaluating the best estimation of afterslip on the Paganica fault is not the goal of this study.

### Comparison with InSAR data

The modelled surface displacements were projected onto the LOS direction and compared with the InSAR data. The first SAR image was acquired 6 days after the mainshock; thus, we take April 12, 2009 as the model’s reference epoch. All the displacement profiles and time series are referenced to this date. Consequently, in our results, the “postseismic” displacements refer to April 12, and the “coseismic” displacement includes the 6 days following the mainshock. The choices of the LOS reference system and reference epoch allow a one-to-one comparison between the model and the InSAR data.

The modelled LOS profiles compare well with the coseismic InSAR data (Fig. 6a), thus confirming that the model geometry and parameters are appropriate. Both the ELA and PLA constitutive models equally fit the data, indicating that the amount of plastic deformation in the earliest 6 days is negligible with respect to the coseismic deformation. Moreover, the root mean-square errors (RMSEs) of the ELA and PLA models (20–30 mm) (Fig. 6b) are similar to those of coseismic inversions of InSAR data^19^ but are greater than those of joint inversions of GPS and InSAR data^28^.

In the postseismic phase, the models include contributions from linear afterslip and plastic deformation under gravity loading. The PLA model captures almost all the features of the InSAR data (Fig. 6a). Indeed, at the Mt Ocre ridge, the PLA model reproduces the data well, including the observed convexity in the displacement profile. The convexity (i.e., the excess displacement at Mt Ocre with respect to the L’Aquila plain) indicates that the plastic deformation that is induced by gravity is a more important process than the afterslip. In contrast, the ELA model does not reproduce the data at Mt Ocre, indicating that plastic deformation, which is absent in the ELA, dominates in areas with high relief energy. In fact, the PLA model is significantly more accurate at Mt Ocre; it is characterised by RMSEs that are approximately half those of the ELA model (5–6 mm versus 9–11 mm; Fig. 6b). Several discrepancies between the model displacement and the data are located near the Paganica fault in both the ELA and PLA models (the red arrow on section A, Fig. 6a). Here, the absence of topographic relief confirms that the observed InSAR displacement is due to unmodelled shallow postseismic slip.

**Figure 5:**
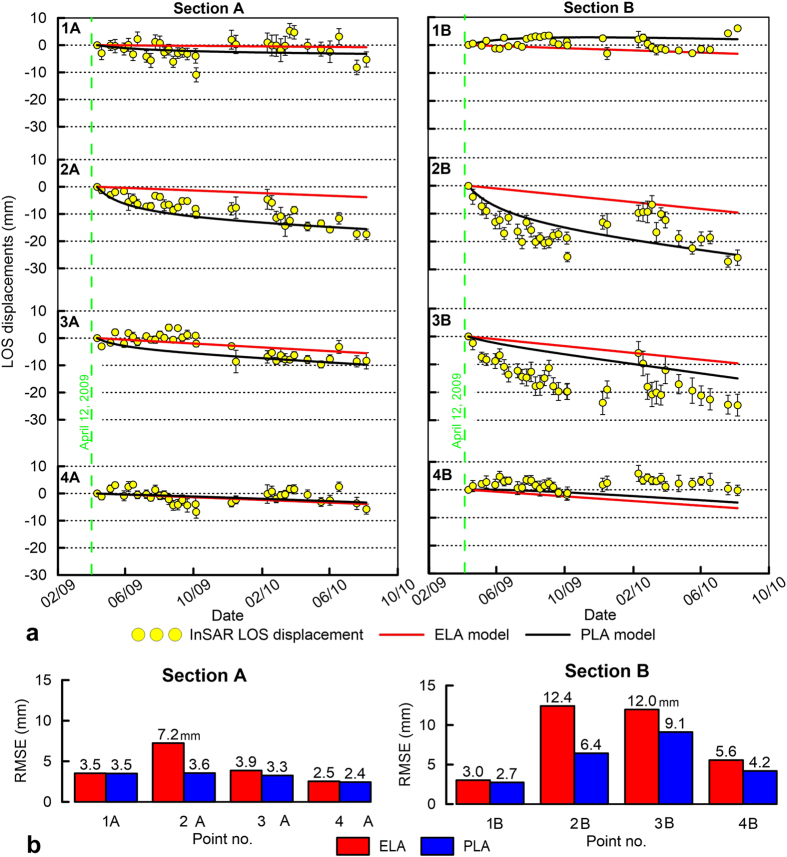
(**a**) Time series of the observed (yellow circles) and modelled (red and black lines) LOS displacement time series for points on sections A and B in Fig. 3a. The black line represents the PLA model results, and the red line represents the ELA model results. The error bars refer to the standard deviation among the point scatterers inside a circular area with a 100 m radius. **(b**) RMSE between observed and modelled displacements for the ELA and PLA models. Figures were created with Golden Software Grapher.

To enhance the role of gravity, we selected points at different topographic heights and discussed the observed and simulated postseismic time series of the LOS displacements at these points (Fig. 5).

For section A, the ELA model displacements in the high-relief zones near the Mt Ocre ridge (point 2A) are linear and do not fit the observed time series. Instead, the PLA model displacements exhibit a nonlinear trend for the first 3–4 months, similar to the trend in the observed data (Fig. 5a). This nonlinear trend cannot be only associated with the shallow afterslip of the main fault; in fact, the Mt Ocre ridge is located more than 4 km from the aftershock cloud (Fig. 2a), which is more than the thickness of the model’s plastic layer.

On flat or gently sloping ground, the observed and modelled displacement time series are comparable, mostly linear, and coherent with the lack of relief and the proximity of the main fault (points 3A and 4A). The ELA and PLA models produce almost identical results because the deformation is primarily driven by the afterslip of the Paganica fault. In the farthest locations (point 1A), the postseismic displacements are negligible despite the topographic height because these locations are not affected by the instabilities introduced by the mainshock.

Points 1B and 4B in section B, which are not located on Mt Ocre ridge, exhibit linear trends that are similar to those of points 1A and 4A. The ELA model is not able to fit the LOS displacement at point 2B on the Mt Ocre ridge, and the PLA model underestimates the observed trend. Point 2A has RMSEs of the ELA and PLA models that are double those at point 2B (Fig. 5b), which indicates that both the plastic deformation and the afterslip contribute to the observed deformation. In fact, the residual of the PLA model can be reduced by assuming a non-linear downward afterslip velocity. The ELA and PLA models also do not match the LOS displacements at point 3B (Fig. 5a,b). The deformation at this location is primarily caused by the tectonic afterslip that moves the wedge downward, possibly at an exponential velocity that decreases with time.

The effect of deep gravitational processes to the surface can be understood from the displacement pattern at depth in the PLA model (Fig. 7). Here, the displacement computed 90 days after the mainshock is highest at the Mt Ocre ridge and is approximately ten times higher than the displacements on the L’Aquila plain. This excess displacement is related to the plastic strain accumulation near the relief due to the yielding of the material.

The displacement vectors to the right of the Mt Ocre ridge’s axis are mainly directed NE towards the L’Aquila plain. Conversely, the vectors to the left of the ridge axis are rotated towards the SW to recover the elastic strains induced by the coseismic slip. The model’s displacement pattern is coherent with gravitational spreading triggered by the earthquake.

## Discussion

We investigated the postseismic effects of the April 6, 2009 L’Aquila earthquake through the numerical modelling of SAR data. We proved that the elastoplastic behaviour of the material under gravitational loading satisfactorily describes the postseismic displacement observed by SAR in the 16 months following the mainshock. The earthquake changed the *in situ* stress state and the topography. The coseismic subsidence also reduced the lateral constraint at the base of the Mt Ocre ridge. The gravity load was then able to generate postseismic deformation whose shape and temporal evolution depend on the rheological characteristics and the mass distribution at shallow depths (0–3 km).

Previous studies modelled the postseismic displacement based only on afterslip^22^,^28^,^29^, thus neglecting the effects of non-tectonic phenomena. In fact, the non-tectonic forces are significant in the postseismic phase^35^. In the study area, several phenomena, such as groundwater flow, liquefaction, or gravitational processes, may have been responsible for postseismic ground settlement or uplift. In particular, the Upper Pleistocene-Holocene sackung-type landforms on the Mt Ocre ridge resulted from gravitational processes possibly triggered by seismic activity^37^,^40^. These landforms, typically attributed to gravity, are located in the same area where the SAR detected excess postseismic displacement with respect to the movement caused solely by fault slip. We hypothesized that if the landforms had a gravitational origin, the postseismic displacement around Mt Ocre may also have been affected by gravity.

To verify this hypothesis, a 2D finite element analysis of two sections crossing the Mt Ocre ridge was performed. This technique takes advantage of the geometrical symmetry of the displacement. Two different analyses have been conducted to compare the elastic behaviour with the elastoplastic behaviour of the involved materials. The numerical model based on pure elasticity is unable to fit the observed displacement patterns and time series. Conversely, the elastoplastic model satisfactorily describes the displacement time series. This result implies that the nonlinearity in the displacement depends on the plastic strain accumulation in the yielded material along the Mt Ocre ridge.

**Figure 6:**
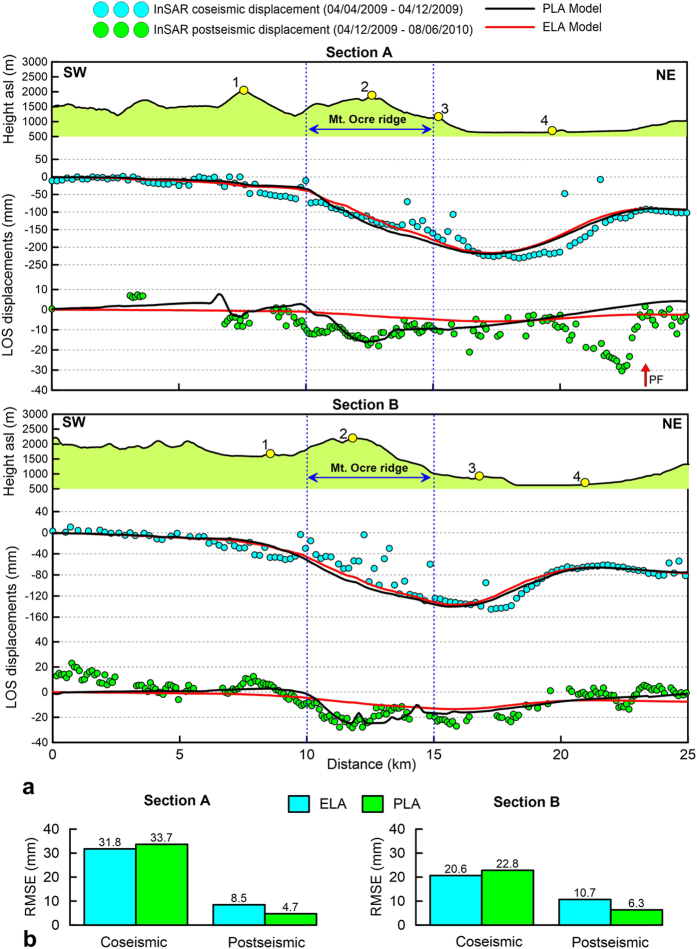
(**a**) Observed (dots) and modelled (black and red lines) LOS CSK displacements between April 4 – April 12, 2009 (cyan dots) and April 12, 2009 – August 06, 2010 (green dots) for sections A and B. (**b**) RMSE of ELA and PLA models for sections A and B. The coseismic RMSE is evaluated along the whole section, the postseismic RMSE is evaluated between 10 and 15 km. Figures were created with Golden Software Grapher.

Given the homogeneous model material and the two-dimensional analysis, a first-order comparison between the predictions and data can be made at Mt Ocre. The RMSEs of the PLA model are approximately half those of the ELA model (Figs 5b and 6b), clearly favouring the elastoplastic hypothesis. The geometric symmetry between the topography and the postseismic displacement pattern supports the choice of developing two bidimensional model sections across the Mt Ocre ridge and the L’Aquila plain. The homogeneous model assumption allows us to discriminate between two end-member hypotheses, provided that reasonable model parameters are used.

**Figure 7:**
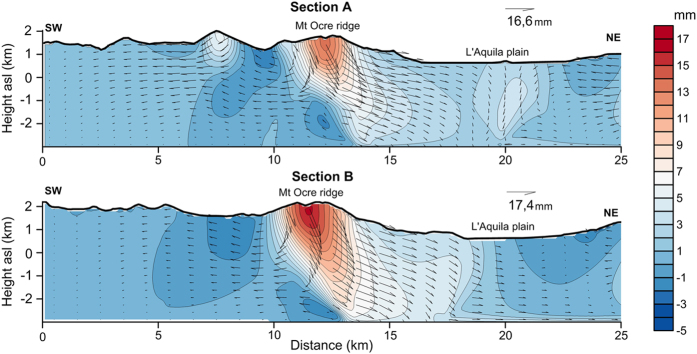
Contour map and vectors of the postseismic displacement field at depth computed three months after the mainshock. Figures were created with Golden Software Surfer.

The changes in the coseismic stress and geometry destabilized Mt Ocre’s relief, causing excess displacements with respect to the L’Aquila plain. In fact, the Mt Ocre ridge is very close to the coseismic subsidence area (Fig. 2a). Once the relief was destabilized, the postseismic stress conditions were able to reactivate pre-existing discontinuities and produce gravitational spreading (Fig. 7). In contrast, the soils in the flat areas are not highly susceptible to groundwater oscillation or liquefaction. Consequently, the displacement in the L’Aquila plain was mainly induced by the afterslip, as confirmed by both the ELA and PLA models (Fig. 6).

During the postseismic period, at least two distinct processes are known to have produced movement in the crust. The first consists of additional slip, usually minor, along the fault, some of which is related to earthquake aftershocks on the ruptured fault. The second process includes visco-plastic flow in the deeper crust. This flow originates from the changes in the stress field following large earthquakes and helps to re-equalize pressure within the crust.

We suggested a third postseismic mechanism in the presence of high topographic gradients: plastic deformation at shallow depths driven by gravitational loading. The amount of postseismic plastic deformation increases with the topographic gradient and ductility of the geomaterials. In our study, the effect of plasticity prevails over the tectonic deformation. Studies that determine the fault afterslip at shallow depths should incorporate plastic behaviour. When performing inversions within purely elastic media, a decoupled plastic analysis must be included in the published study, similar to those already in use at the deeper crustal scale^48^. The adoption of a homogeneous isotropic plastic model allows the postseismic deformation to also be modelled in areas with little geologic information. In general, shallow plastic deformation must be appropriately included when modelling postseismic data close to the fault line.

A similar reasoning applies to the analysis of long-term geologic data located near or on a seismogenic fault, especially when these data are used for regional or site-specific seismic hazard estimates. In this case, determining quantities such as the long-term tectonic fault slip rate or the slip per event requires quantifying the plastic deformation at shallow depths and possibly other non-tectonic phenomena. Failing to do so will introduce unknown biases in the seismic hazard analyses.

In conclusion, the postseismic deformation of the 2009 L’Aquila earthquake is partly caused by the plastic behaviour and reactivation of pre-existing discontinuities under gravitational loading and coseismic stress changes. This outcome is based on the net difference between the pure elastic and elastoplastic displacements.

## Methods

### PSP processing of the COSMO-SkyMed X-band SAR data

For this study, a set of 35 Cosmo-SkyMed SAR acquisitions was collected. All the images are in stripmap-image acquisition mode, with a resolution of 3 m x 3 m per pixel, HH polarization, a nominal incidence angle of 40 ° and a maximum baseline of 1,489 m.

All the images were processed by applying the Persistent Scatterer Pair (PSP) technique. This approach was recently proposed^49^,^50^,^38^ to identify persistent scatterers (intended in the general sense of scatterers that exhibit interferometric coherence for the time period and baseline span of the acquisitions, including both point-like and distributed scatterers) in series of full-resolution SAR images and to retrieve the corresponding elevations and displacements.

Unlike standard persistent scatterer interferometry techniques, the PSP method relies only on the relative signal in pairs of nearby points, both for the identification and analysis of persistent scatterers (PSs, hence the name PSP). For this reason, the PSP technique is intrinsically robust to spatially correlated disturbances (such as atmospheric and orbital artefacts) and does not require preliminary processing to compensate for these contributions to the signal. In addition, deviations from the displacement evolution models typically used in PS techniques (for example, due to strong nonlinear evolutions) are mitigated in the PSP approach because close points likely have similar deviations.

However, working with arcs requires many more computations than analysing single points; in fact, N points can form (N-1)N/2 different arcs. The PSP approach is based on a complex algorithm designed to find a set of valid (in the sense of the multi-acquisition coherence or analogous parameters) arcs to form a graph with a given connectivity and cardinality. The PSs are identified as the nodes of this graph.

A high number of arcs and a high connectivity of the graph guarantee reliable PS identification and accurate PS measurements. Moreover, these factors allow coherent information to be fully extracted even from low-intensity SAR signals, even when strongly scattering structures are not present, the observed signal is weak or the scattering is distributed, as in the case of rather smooth surfaces or natural terrains. Thus, the PSP algorithm does not rely on filtering that involves a loss of resolution, such as that recently introduced for analysing distributed scatterers^51^, and instead exploits the redundancy of the pair-of-point connections, thereby maintaining the full resolution of the original SAR images.

In this sense, PSs are intended in the general sense of scatterers whose backscattering properties remain unaltered over time (at least for the period of interest), as the name suggests, but can also be fully distributed. In fact, due to the high resolution and stricter orbital control, the interferometric baseline in recent satellite SAR systems is below the critical limit, and no strong spatial decorrelation phenomena are present.

The redundant information provided by the highly connected PSP graph is conveniently exploited to guarantee more accurate measurements in a recently published method for robust phase unwrapping and finite difference integration^52^, two key processing steps in the reconstruction of PS elevations and displacements from interferometric SAR data. The use of a multi-scale decomposition method^53^ allows us to perform a global processing approach for large areas with high PS densities, despite the high computational demand required by the exploitation of very redundant information.

The advantages of globally exploiting redundant arcs connecting different pairs of PSs are particularly relevant for obtaining reliable and accurate measurements when the PSs are very sparse, as in the considered region of L’Aquila, which is characterized by the presence of mountains and large vegetated areas (supplementary Fig. S1).

### Accuracy of the COSMO-SkyMed PSP measurements

The PSP SAR interferometry method has been validated in different test sites and used in several operational productions for several types of applications and with different types of SAR data^38^. In particular, the characteristics of the COSMO-SkyMed satellite SAR constellation are very suitable for SAR interferometry and PSP processing. The short wavelength and low noise level of the COSMO-SkyMed SAR system make it possible to collect a significant backscattering signal from rather smooth surfaces without strong scattering structures and render millimetric displacements in interferometric measurements significant. The baselines between the different acquisitions are kept below the critical limit for interferometry but sufficiently large (up to 2 km) to guarantee a metric sensitivity to elevation. This sensitivity, coupled with the fine ground resolution, allow measurements to be very precisely located in 3D space. The fine ground resolution allows the collection of a coherent backscattering signal even from small spots of bare soil in vegetated terrains.

The tests performed with COSMO-SkyMed data proved that very dense (up to several tens or hundreds of thousands per square kilometre) PSP measurements, characterized by metric localization and millimetric displacement accuracies, are obtained in all type of scenes in which a coherent, even if low, backscattering signal is expected (practically excluding only densely vegetated and cultivated terrains, where the signals backscattered at different times are not coherent). In particular, the results^38^ show that the standard deviation of the deformation measurement error is on the order of 2 mm, typically ranging between 1 and 3 mm depending on the quality of the specific PS and the type of ground cover, whereas the statistics for the height measurements show a standard deviation of approximately 0.7 m. The measurement densities are typically several tens of thousands per square kilometre and greater than 100,000 PS/km^2^ in the most urbanized areas.

### Geomechanical setting of Mt Ocre ridge

The geological and geometric setting of Mt. Ocre ridge is complex. The Mt. Ocre area is typical of the central Apennines; it is characterised by a jointed carbonate rock mass that is superimposed on a flysch stratum. The geometric relationship between the carbonates and the flysch has been widely investigated in the surrounding areas using geomorphological, seismic, and borehole data^42^,^43^,^44^,^54^. The carbonate rocks are present at most of the investigated depths (Fig. 1b), while the claystone and sandstone flysch formation is relatively thin (<400 m). In addition, the flysch deposits are mainly lithified and are mostly located at depths of approximately 2–3 km; thus, they have stiffness and strength characteristics that are similar to those of the carbonates^46^. All of these factors suggest that the carbonate and flysch layers have comparable mechanical properties at the time scale of a few months, which is of interest for this study.

The study area contains a highly-jointed carbonate rock mass with mapped fractures that are significantly less than l km long^40^. Thus, we based the numerical modelling on an equivalent continuum approach^47^,^55^,^56^. The discontinuous rock mass can be considered to be homogeneous and continuous, and the equivalent stiffness and strength parameters can be derived from the geomechanical properties of both the intact rock and the joints. The equivalent continuum approach was chosen for two reasons: i) the individual rock mass discontinuities could not be considered at the scale of the investigated phenomenon (the model is 30 km long and approximately 4 km thick); and ii) at this scale, it is not convenient to analyse the mechanical behaviour of the intact rock–joint system in a discontinuous mode. Thus, the stiffness and strength parameters of the study area can be modelled as homogeneous and isotropic.

### Modelling

The 2D finite-element models were developed using the commercial software MSC Marc 2013^57^. The models represent two sections (A and B) that are 30 km long and approximately 4 km deep (Fig. 4b). The sections are meshed with eight-node, isoparametric arbitrary quadrilateral plane strain elements (6930 elements for section A and 6301 for section B). The spatial resolution of the grid is 100 m close to the surface, and the element dimension increases toward the lateral and bottom edges of the domain to values of 200 m. The topographic surface is obtained from a Digital Elevation Model with a resolution of 5 m × 5 m. The material is uniform and isotropic and described by elastic (ELA) and elastic perfectly plastic Mohr-Coulomb (PLA) constitutive models for the two modelled sections. The Mohr-Coulomb failure criterion is defined according to the following relation:


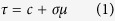


where *τ* is the shear strength, *σ* is the normal stress, c is the cohesion (i.e., the intercept of the failure envelope with the *τ* axis) and *μ* is a friction coefficient (which is equivalent to tan*ϕ*, where *ϕ* is the angle of internal friction). Compression is assumed to be positive. The Mohr-Coulomb model presents a non-associated flow rule.

In the generalised Mohr-Coulomb model that was implemented in the MARC software, the deviatoric yield function is assumed to be a linear function of the hydrostatic stress according to equation (2):


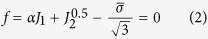


where J_1_ and J_2_ are the stress invariants, and α and 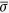 are material-dependent constants.

The constants α and 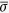 in equation (2) can be related to the cohesion c and the friction angle ϕ by:


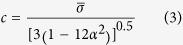



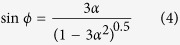


The elastic parameters were chosen based on data from the literature^25^. The strength parameters were selected after a trial and error analysis to reproduce the observed displacements (Table 1).

Parametric analyses showed that a one order of magnitude change in the cohesion does not significantly affect the modelled displacements because it only causes a shift in the Mohr-Coulomb failure envelope. Conversely, the obtained deformation profiles depend heavily on the friction coefficient, especially at high confinement stress levels.

Our strength parameters are within the typical ranges of the equivalent rock-strength parameters that were used in studies of rock mass deformations in nearby areas of central Italy^55^,^56^,^58^. The friction angle (40°) is consistent with laboratory data and is typical of jointed carbonate rocks in the central Apennines. The cohesion (160 kPa) is lower than typical values from laboratory tests. However, cohesion is a scale-dependent parameter^59^; laboratory values are not representative of rock mass cohesion, which is often one order of magnitude lower.

A uniform density of *ρ* = 2500 kg/m^3^ is assumed for all the elements. The numerical model is subjected to a constant gravitational acceleration (9.81 m/s^2^) applied as a body force. The analyses have been conducted in terms of total stresses, neglecting the presence of subsurface water.

The position and length of the red wedge (Fig. 4b) have been defined on the right by considering the depth projection of the Paganica fault. On the left, the length and dip of the wedge have been imposed according to the cloud of hypocentres at depth (supplementary Fig. S3) and by best fitting the observed coseismic displacement profiles.

### Misfit calculation

The misfit between the observed and predicted displacements is evaluated using the root-mean-square error (RMSE):


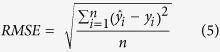


where 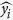 and y_i_ are the predicted and observed values, respectively, and *n* is the number of observations.

The RMSEs of the displacement profiles (Fig. 6a) are evaluated along a 25-km-long section for the coseismic phase and between 10 and 15 km for the postseismic phase.

The RMSE of the displacement time series is evaluated as the square root of the mean of the squares of the deviations between the measured and predicted displacements at the same observation moment.

## Additional Information

**How to cite this article**: Albano, M. *et al.* Gravity-driven postseismic deformation following the Mw 6.3 2009 L’Aquila (Italy) earthquake. *Sci. Rep.* **5**, 16558; doi: 10.1038/srep16558 (2015).

## Supplementary Material

Supplementary Figures

## Figures and Tables

**Table 1:** Model parameters.

Parameter	Name	Symbol	Value
Elastic parameters	Young’s modulus	E	5e10 Pa
	Poisson ratio	*ν*	0.2
Strength parameters	Cohesion	c	160 kPa
	Friction angle	ϕ	40°
State parameters	Density	*ρ*	2500 kg/m^3^

## References

[b1] Doglioni C. Geological remarks on the relationships between extension and convergent geodynamic settings. Tectono- physics 252, 253–267 (1995).

[b2] Meletti C., Patacca E. & Scandone P. Construction of a Seismotectonic Model: The Case of Italy. Pure Appl. Geophys. 157, 11–35 (2000).

[b3] Keller J., Minelli G. & Pialli G. Anatomy of late orogenic extension: The Northern Apennines case. Tectonophysics 238, 275–294 (1994).

[b4] Galadini F. & Galli P. Paleoseismology of silent faults in the Central Apennines (Italy): The Mt. Vettore and Laga Mts. faults. Ann. Geophys. 46, 815–836 (2003).

[b5] Mariucci M. T., Montone P. & Pierdominici S. Present-day stress in the surroundings of 2009 L’Aquila seismic sequence (Italy). Geophys. J. Int. 182, 1096–1102 (2010).

[b6] Devoti R., Esposito A., Pietrantonio G., Pisani A. R. & Riguzzi F. Evidence of large scale deformation patterns from GPS data in the Italian subduction boundary. Earth Planet. Sci. Lett. 311, 230–241 (2011).

[b7] Galli P., Galadini F. & Pantosti D. Twenty years of paleoseismology in Italy. Earth-Science Rev. 88, 89–117 (2008).

[b8] Chiarabba C. *et al.* The 2009 L’Aquila (central Italy) M W 6.3 earthquake: Main shock and aftershocks. Geophys. Res. Lett. 36, L18308 (2009).

[b9] Chiaraluce L. *et al.* The 2009 L’Aquila (central Italy) seismic sequence. Boll. di Geofis. Teor. e Appl. 52, 367–387 (2011).

[b10] Carafa M. M. C. & Barba S. The stress field in Europe: optimal orientations with confidence limits. Geophys. J. Int. 193, 531–548 (2013).

[b11] Meletti C., Patacca E. & Scandone P. Il sistema compressione-distensione in Appennino. *Cinquanta Anni di Attività Didatt. e Sci. del Prof*. Felice Ippolito 361–370 (1995).

[b12] DISS Working Group. Database of Individual Seismogenic Sources (DISS), Version 3.1.1: A compilation of potential sources for earthquakes larger than M 5.5 in Italy and surrounding areas (2010). Available at: http://diss.rm.ingv.it/diss/ (Accessed: 16th October 2014).

[b13] Rovida A., Camassi R., Gasperini P. & Stucchi M. CPTI11, the 2011 version of the Parametric Catalogue of Italian Earthquakes (2011). Available at: http://emidius.mi.ingv.it/CPTI11/ (Accessed: 16th October 2014).

[b14] Galadini F. & Galli P. Active Tectonics in the Central Apennines (Italy) – Input Data for Seismic Hazard Assessment. Nat. Hazards 22, 225–268

[b15] Falcucci E. *et al.* The Paganica Fault and Surface Coseismic Ruptures Caused by the 6 April 2009 Earthquake (L’Aquila, Central Italy). Seismol. Res. Lett. 80, 940–950 (2009).

[b16] Gori S. *et al.* Constraining primary surface rupture length along the Paganica fault (2009 L’Aquila earthquake) with geological and geodetic (DInSAR and GPS) data. Ital. J. Geosci. 131, 359–372 (2012).

[b17] EMERGEO WORKING GROUP. Evidence for surface rupture associated with the Mw 6.3 L’Aquila earthquake sequence of April 2009 (central Italy). Terra Nov. 22, 43–51 (2010).

[b18] Moro M. *et al.* Historical earthquakes and variable kinematic behaviour of the 2009 L’Aquila seismic event (central Italy) causative fault, revealed by paleoseismological investigations. Tectonophysics 583, 131–144 (2013).

[b19] Atzori S. *et al.* Finite fault inversion of DInSAR coseismic displacement of the 2009 L’Aquila earthquake (central Italy). Geophys. Res. Lett. 36, (2009).

[b20] ISIDe Working Group. ISIDe - Italian Seismological Instrumental and Parametric Data-Base (2010). Available at: http://iside.rm.ingv.it/iside/standard/index.jsp (Accessed: 16th October 2014).

[b21] Amato A. & Galli P. Introducing the special issue on the 2009 L’Aquila earthquake. Boll. di Geofis. Teor. e Appl. 52, 357–365 (2011).

[b22] Cheloni D. *et al.* Coseismic and initial post-seismic slip of the 2009 M w 6.3 L’Aquila earthquake, Italy, from GPS measurements. Geophys. J. Int. 181, 1539–1546 (2010).

[b23] Walters R. J. *et al.* The 2009 L’Aquila earthquake (central Italy): A source mechanism and implications for seismic hazard. Geophys. Res. Lett. 36, L17312 (2009).

[b24] Serpelloni E., Anderlini L. & Belardinelli M. E. Fault geometry, coseismic-slip distribution and Coulomb stress change associated with the 2009 April 6, Mw 6.3, L’Aquila earthquake from inversion of GPS displacements. Geophys. J. Int. 188, 473–489 (2012).

[b25] Trasatti E., Kyriakopoulos C. & Chini M. Finite element inversion of DInSAR data from the Mw 6.3 L’Aquila earthquake, 2009 (Italy). Geophys. Res. Lett. 38, (2011).

[b26] Volpe M., Piersanti A. & Melini D. Complex 3-D Finite Element modelling of the 2009 April 6 L’Aquila earthquake by inverse analysis of static deformation. Geophys. J. Int. 188, 1339–1358 (2012).

[b27] Amoruso A., Barba S., Crescentini L. & Megna A. Inversion of synthetic geodetic data for dip-slip faults: clues to the effects of lateral heterogeneities and data distribution in geological environments typical of the Apennines (Italy). Geophys. J. Int. 192, 745–758 (2012).

[b28] D’Agostino N., Cheloni D., Fornaro G., Giuliani R. & Reale D. Space-time distribution of afterslip following the 2009 L’Aquila earthquake. J. Geophys. Res. 117, B02402 (2012).

[b29] Cheloni D. *et al.* Coseismic and post-seismic slip of the 2009 L’Aquila (central Italy) MW 6.3 earthquake and implications for seismic potential along the Campotosto fault from joint inversion of high-precision levelling, InSAR and GPS data. Tectonophysics 622, 168–185 (2014).

[b30] Gualandi A., Serpelloni E. & Belardinelli M. E. Space-time evolution of crustal deformation related to the Mw 6.3, 2009 L’Aquila earthquake (central Italy) from principal component analysis inversion of GPS position time-series. Geophys. J. Int. 197, 174–191 (2014).

[b31] Pollitz F. F. Mantle Flow Beneath a Continental Strike-Slip Fault: Postseismic Deformation After the 1999 Hector Mine Earthquake. Science 293, 1814–1818 (2001). 11546869 10.1126/science.1061361

[b32] Jo´nsson S., Segall P., Pedersen R. & Bjo¨rnsson G. Post-earthquake ground movements correlated to pore-pressure transients. Nature 424, 179–183 (2003). 12853953 10.1038/nature01776

[b33] Stramondo S. *et al.* Surface movements in Bologna (Po Plain — Italy) detected by multitemporal DInSAR. Remote Sens. Environ. 110, 304–316 (2007).

[b34] Chini M. *et al.* Coseismic liquefaction phenomenon analysis by COSMO-SkyMed: 2012 Emilia (Italy) earthquake. Int. J. Appl. Earth Obs. Geoinf. 39, 1–14 (2015).

[b35] Lacroix P., Perfettini H., Taipe E. & Guillier B. Coseismic and postseismic motion of a landslide: Observations, modeling, and analogy with tectonic faults. Geophys. Res. Lett. 41, 6676–6680 (2014).

[b36] Moro M., Saroli M., Tolomei C. & Salvi S. Insights on the kinematics of deep-seated gravitational slope deformations along the 1915 Avezzano earthquake fault (Central Italy), from time-series DInSAR. Geomorphology 112, 261–276 (2009).

[b37] Moro M. *et al.* Analysis of large, seismically induced, gravitational deformations imaged by high-resolution COSMO- SkyMed synthetic aperture radar. Geology 39, 527–530 (2011).

[b38] Costantini M. *et al.* Persistent Scatterer Pair Interferometry: Approach and Application to COSMO-SkyMed SAR Data. IEEE J. Sel. Top. Appl. Earth Obs. Remote Sens. 7, 2869–2879 (2014).

[b39] Radbruch-Hall D. H., Varnes D. J. & Savage W. Z. Gravitational spreading of steep-sided ridges (“sackung”) in Western United States. Bull. Int. Assoc. Eng. Geol. 13, 23–35 (1976).

[b40] Salvi S. *et al.* Investigation of the active Celano-L’Aquila fault system, Abruzzi (central Apennines, Italy) with combined ground-penetrating radar and palaeoseismic trenching. Geophys. J. Int. 155, 805–818 (2003).

[b41] Valoroso L. *et al.* Radiography of a normal fault system by 64,000 high-precision earthquake locations: The 2009 L’Aquila (central Italy) case study. J. Geophys. Res. Solid Earth 118, 1156–1176 (2013).

[b42] Mostardini F. & Merlini S. Appennino centro meridionale: sezioni geologiche e proposta di modello strutturale. Mem. della Soc. Geol. Ital. 35, 177–202 (1986).

[b43] Vezzani L., Festa A. & Ghisetti F. C. in Geol. Soc. Am. Special Paper 469, 1–58 (2010).

[b44] Satolli S. & Calamita F. Differences and similarities between the central and the southern Apennines (Italy): Examining the Gran Sasso versus the Matese-Frosolone salients using paleomagnetic, geological, and structural data. J. Geophys. Res. Solid Earth 113, 1–16 (2008).

[b45] Bonini L., Di Bucci D., Toscani G., Seno S. & Valensise G. On the complexity of surface ruptures during normal faulting earthquakes: excerpts from the 6 April 2009 L’Aquila (central Italy) earthquake (Mw 6.3). Solid Earth 5, 389–408 (2014).

[b46] Chiarabba C., Bagh S., Bianchi I., De Gori P. & Barchi M. Deep structural heterogeneities and the tectonic evolution of the Abruzzi region (Central Apennines, Italy) revealed by microseismicity, seismic tomography, and teleseismic receiver functions. Earth Planet. Sci. Lett. 295, 462–476 (2010).

[b47] Sitharam T. G., Sridevi J. & Shimizu N. Practical equivalent continuum characterization of jointed rock masses. Int. J. Rock Mech. Min. Sci. 38, 437–448 (2001).

[b48] Pollitz F. F., Nyst M., Nishimura T. & Thatcher W. Inference of postseismic deformation mechanisms of the 1923 Kanto earthquake. J. Geophys. Res. 111, B05408 (2006).

[b49] Costantini M., Falco S., Malvarosa F. & Minati F. A New Method for Identification and Analysis of Persistent Scatterers in Series of SAR Images. in IGARSS 2008 - 2008 IEEE International Geoscience and Remote Sensing Symposium 2, II–449–II–452 (2008).

[b50] Costantini M., Minati F., Trillo F. & Vecchioli F. Enhanced PSP SAR interferometry for analysis of weak scatterers and high definition monitoring of deformations over structures and natural terrains. in 2013 IEEE International Geoscience and Remote Sensing Symposium - IGARSS 876–879 (2013).

[b51] Ferretti A. *et al.* A New Algorithm for Processing Interferometric Data-Stacks: SqueeSAR. IEEE Trans. Geosci. Remote Sens. 49, 3460–3470 (2011).

[b52] Costantini M., Malvarosa F. & Minati F. A General Formulation for Redundant Integration of Finite Differences and Phase Unwrapping on a Sparse Multidimensional Domain. IEEE Trans. Geosci. Remote Sens. 50, 758–768 (2012).

[b53] Costantini M., Malvarosa F., Minati F. & Vecchioli F. Multi-scale and block decomposition methods for finite difference integration and phase unwrapping of very large datasets in high resolution SAR interferometry. in 2012 IEEE International Geoscience and Remote Sensing Symposium 5574–5577 (2012).

[b54] Finetti I. R. CROP PROJECT: Deep Seismic Exploration of the Central Mediterranean and Italy. (Elsevier, 2005).

[b55] Esposito C., Martino S. & Mugnozza G. S. Mountain slope deformations along thrust fronts in jointed limestone: An equivalent continuum modelling approach. Geomorphology 90, 55–72 (2007).

[b56] Bianchi Fasani G., Di Luzio E., Esposito C., Martino S. & Scarascia-Mugnozza G. Numerical modelling of Plio-Quaternary slope evolution based on geological constraints: a case study from the Caramanico Valley (Central Apennines, Italy). Geological Society, London, Special Publications 351, 201–214 (2011).

[b57] MSC Software Corporation. MARC 2013 Volume A: Theory and user information. (2013) Available at: https://simcompanion.mscsoftware.com/infocenter/index?page=content&id=DOC10338&cat=MARC_DOCUMENTATION_2013&actp=LIST. (Accessed: 5th November 2013).

[b58] Martino S., Moscatelli M. & Scarascia Mugnozza G. Quaternary mass movements controlled by a structurally complex setting in the central Apennines (Italy). Eng. Geol. 72, 33–55 (2004).

[b59] Chemenda A. I., Bois T., Bouissou S. & Tric E. Numerical modelling of the gravity-induced destabilization of a slope: The example of the La Clapière landslide, southern France. Geomorphology 109, 86–93 (2009).

