# Corrigendum: Gravity-driven postseismic deformation following the Mw 6.3 2009 L’Aquila (Italy) earthquake

**DOI:** 10.1038/srep20372

**Published:** 2016-02-04

**Authors:** Matteo Albano, Salvatore Barba, Michele Saroli, Marco Moro, Fabio Malvarosa, Mario Costantini, Christian Bignami, Salvatore Stramondo

Scientific Reports 5: Article number: 1655810.1038/srep16558; published online: 11102015; updated: 02042016

In the original version of this Article, affiliation 1 was incorrectly listed as ‘National Institute of Geophysics and Volcanology, National Earthquake Center, Rome, 00143, Italy’.

The correct affiliation is listed below:

‘Istituto Nazionale di Geofisica e Vulcanologia, Rome, 00143, Italy’

This error has now been corrected in the PDF and HTML versions of the Article.

