# Supplementary material for: Gravity-driven postseismic deformation following the Mw 6.3 2009 L’Aquila (Italy) earthquake
Source: Sci Rep. 2015 Nov 10;5:16558. doi: 10.1038/srep16558 (PMC4639723; doi:10.1038/srep16558)
Supplement: Supplementary Figures [file srep16558-s1.pdf]

Supplementary information for “Gravity-driven postseismic deformation following the Mw 6.3 2009 L’Aquila (Italy) earthquake”

Matteo Albano[1,*], Salvatore Barba[1], Michele Saroli[2], Marco Moro[1], Fabio Malvarosa[3], Mario Costantini[3], Christian Bignami[1], and Salvatore Stramondo[1]

[1]National Institute of Geophysics and Volcanology, National Earthquake Center, Rome, 00143, Italy
[2]University of Cassino and Southern Lazio, Department of Civil and Mechanical Engineering, Cassino, 03043, Italy
[3]e-GEOS, an Italian Space Agency (ASI) and Telespazio Company, Rome, 00156, Italy
[*]matteo.albano@ingv.it

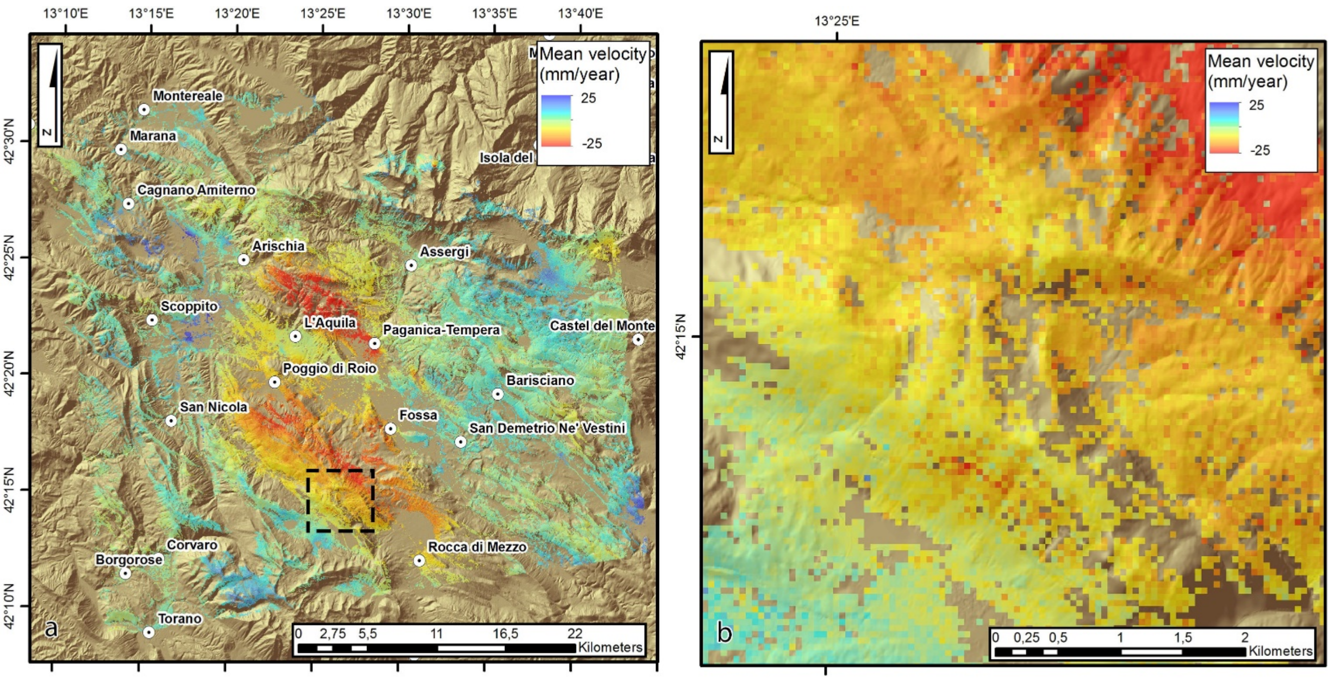

Figure S1. PSP mean velocity measurements obtained from the COSMO-SkyMed Stripmap SAR data (35 ascending acquisitions, April 2009 - August 2010) in the mountainous area of L’Aquila, Italy. The left image shows the entire processed area, corresponding to the COSMO-SkyMed frame extent, and the right image highlights the very dense PSP measurements obtained over grassland and bare soil (black square in Fig. S1a), despite the absence of strong scatterers (PSP measurements are not located in cultivated fields or forests because their backscattering properties change with time). Figures were created with ArcGIS.

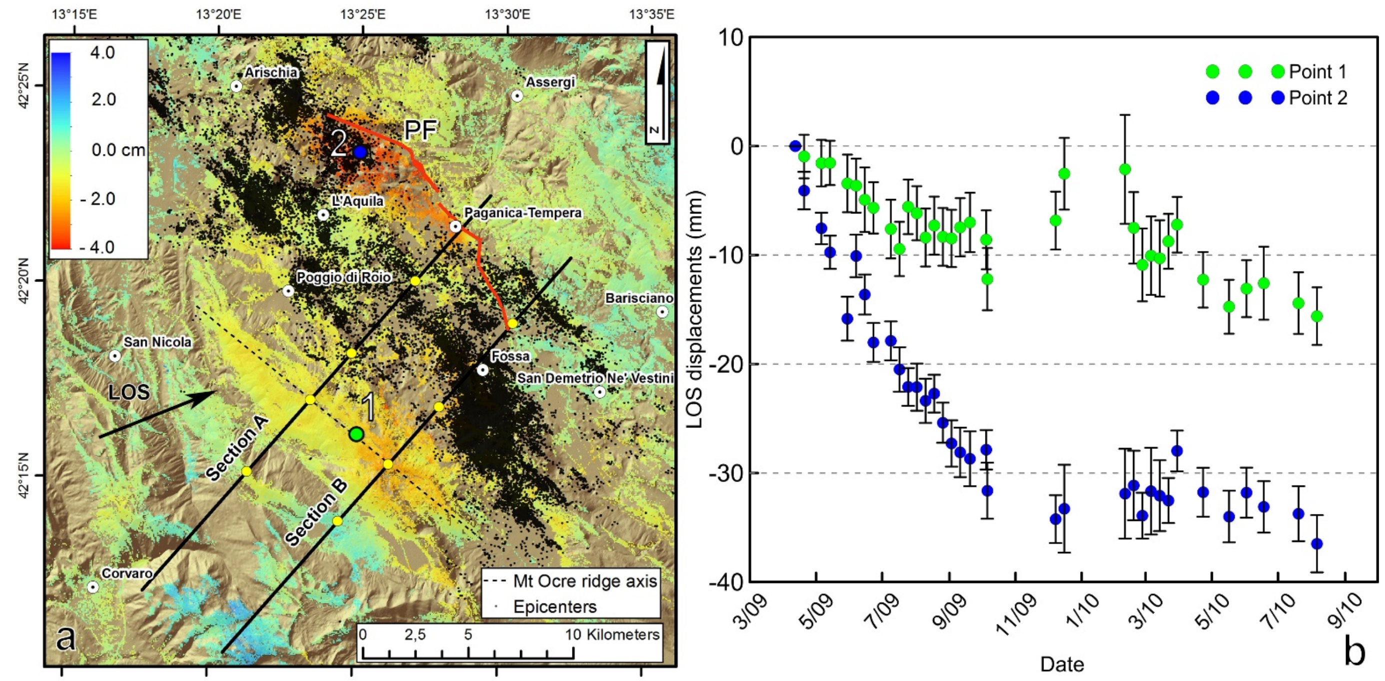

Figure S2. (a) Line-of-sight postseismic displacement map from the InSAR CSK dataset for the time interval between April 12, 2009 and August 6, 2010, together with the position of the aftershocks. (b) LOS displacement time series for points 1 and 2 in Fig. S2a. The yellow circles refer to Fig. 3a. The error bars refer to the standard deviation among the persistent scatterers contained within a circular area with a 500-m radius. Figures were created with ArcGIS and Golden Software Grapher.

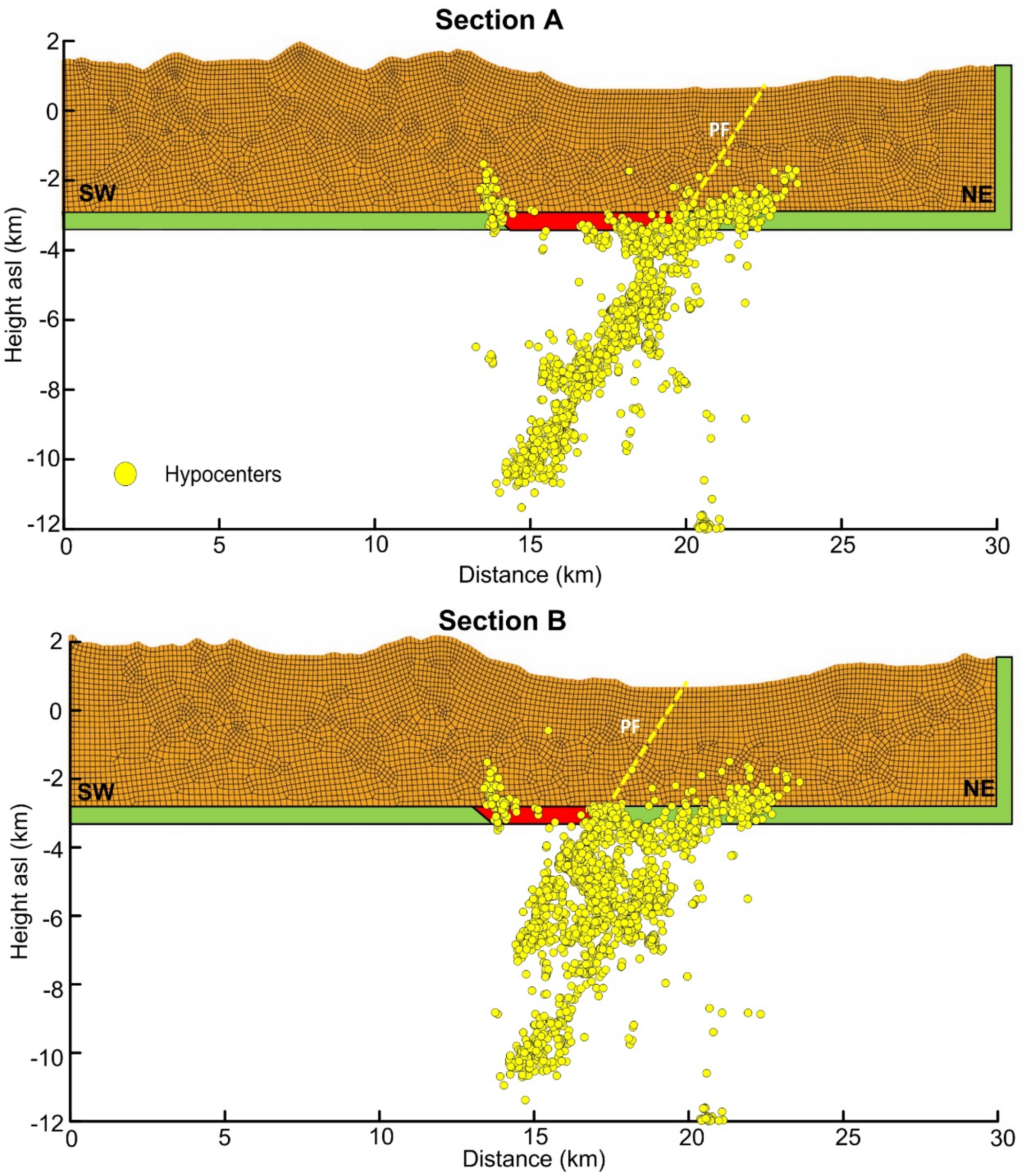

Figure S3. Finite element models for sections A and B in Fig. S2a. The yellow dots indicate the aftershock hypocentres along the two modelled sections. Figure was created with Adobe Photoshop and Golden Software Grapher.
